# The influence and interaction of exposure to pro-smoking and anti-smoking messaging on youth smoking behaviour and susceptibility

**DOI:** 10.18332/tid/114066

**Published:** 2019-11-27

**Authors:** Jingfen Zhu, Jiahui Li, Yaping He, Na Li, Gang Xu, Jinming Yu

**Affiliations:** 1 School of Public Health, Shanghai Jiao Tong University, Shanghai, People’s Republic of China; 2 School of Public Health, Fudan University, Shanghai, People’s Republic of China

**Keywords:** adolescent, tobacco message exposure, current smoking, smoking susceptibility

## Abstract

**INTRODUCTION:**

Exposure to tobacco-related information is an important factor in youth smoking initiation. This study aims to explore the relationship between exposure to pro-smoking and anti-smoking media and adolescents’ current smoking status and susceptibility to smoking, as well as the interaction between exposure to pro-smoking and anti-smoking media information.

**METHODS:**

This cross-sectional study was conducted in 2017. We recruited 12278 students from junior, senior and vocational high schools located in Shanghai, China. The exposure of participants to tobacco promotional and control messages over the past 30 days was examined, as well as current smoking and susceptibility to never smokers’ initiation. Odds ratios (ORs) and 95% confidence intervals (CIs) were estimated using complex samples procedure logistic regression, adjusting for related covariables.

**RESULTS:**

There were 89.3% and 91.5% of adolescents exposed to tobacco pro-smoking and anti-smoking messages. Exposure was more prevalent among males, suburb school and vocational school students. Exposure to pro-tobacco and antitobacco messages, separately, increased and decreased the risk of current smoking and susceptibility to never-smokers’ smoking, respectively, especially among males and junior high school students. Risk was associated with the exposure level (p-trend<0.001). Tobacco control messaging was found to mitigate the influences of tobacco promotion on the risk of both current smoking (AOR=0.64; 95% CI: 0.41–0.99) and susceptibility to smoking (AOR=0.65; 95% CI: 0.46–0.93).

**CONCLUSIONS:**

Exposure to tobacco-related messages was highly prevalent and associated with youth smoking and susceptibility to smoking. It is therefore important to enhance the comprehensiveness and enforcement of promotion bans. Given that tobacco control information can counter the impact of tobacco promotion information on smoking risk, the publicity and dissemination of tobacco control information should be consistently strengthened.

## INTRODUCTION

Tobacco use is one of the major preventable causes of death worldwide^1^. It is a concern that adolescence is a vulnerable period for tobacco addiction, and the age of smoking initiation has decreased in recent years^2^. Previous studies have investigated contributors to adolescent smoking. Apart from personal factors, family environment (such as parental smoking), school environment (peer smoking)^3^ and social environment (such as exposure to media) are also important factors influencing youth smoking behaviours^4^.

The 2012 US Surgeon General’s Report determined a causal association between tobacco promotional advertising and adolescents’ smoking initiation and progression^1^. The marketing strategies of tobacco companies include indirect methods, such as sponsorship of sports events and concerts, as well as direct methods (e.g. billboards and commercials)^5^. Adolescents are commonly exposed to tobacco promotional advertising. For example, in Northern Africa^6^, 90% of adolescents reported seeing smoking on-screen, while 40% and 50% reported observation of a smoking advertisement at a live event or in a magazine, respectively. In addition to traditional forms of tobacco advertising, there has been an increase in advertising exposure on social media sites^7^. A study revealed that young individuals^8^ were regularly exposed to internet-based tobacco advertising through texts, images, and videos^7^. These advertisement and promotion programs not only influence adolescents’ perceived norms, perceived smoking prevalence, and perceptions regarding advantages and disadvantages of smoking^5^, but also indirectly influence adolescents’ smoking risk by affecting the smoking habits of others, involving parents and peers^6^. A positive association between tobacco advertising and youth smoking behaviour has been well established in previous studies^7,9^.

In order to diminish the harmful effects of tobacco promotions on adolescents, various anti-tobacco advertisements have emerged. The themes of the majority of advertisements have emphasised the adverse health effects, resisting social pressures and influences, or the profit motives of the tobacco industry^10^. Several studies^11,12^ examined the impact of anti-tobacco media on the reduction or prevention of tobacco use among youth. Nevertheless, the interaction between exposures to pro-tobacco and anti-tobacco messages has remained elusive. Straub et al.^10^ found that anti-tobacco advertisement may have some preventive effects that cannot fully offset the harmful impacts of pro-tobacco advertisements; however, tobacco control advertising might counteract the effectiveness of cigarette advertising in promoting adolescent smoking behaviour^13^.

Since adolescents are exposed to both pro-tobacco and anti-tobacco advertising simultaneously^14^, the study of this interaction is of great significance. Our study aimed to examine the effects of exposure to both pro-smoking and anti-smoking media on susceptibility to smoking and current smoking as well as their independent impact, and to assess differences in exposure across gender and school types in order to develop targeted interventions.

## METHODS

### Research procedure

This study was conducted from September 2017 to January 2018 through multistage and stratified cluster random sampling. All districts in Shanghai were stratified into urban and suburb areas in the first stage. A total of 33 schools were randomly selected in the second stage according to school type. Under the guidance of a trained investigator, students completed the online questionnaire anonymously in the school’s computer room, and teachers were asked not to be present. Of the 12422 adolescents who participated, 12278 (98.8%) completed the investigation and were included in the analysis.

Informed consent from schools was received with the support of the Shanghai Municipal Education Commission. Participation in this study was voluntary. Written informed consent, provided before enrollment, was obtained from all students, their guardians and school organizers. The informed consent covered objectives, procedures, potential risks and benefits of the study. The study was approved by the Ethics Committee of the Shanghai Jiao Tong University (SJUPN-201703).

### Measures

The questionnaire used in this study was based on the Global Youth Tobacco Survey (GYTS), which was previously assessed to have sufficient validity and reliability^6^.

### Dependent variables

Participants were asked questions in the form: ‘Have you ever tested cigarette smoking (even one or two puffs)?’ and ‘How many days have you smoked in the past 30 days?’. Those participants who reported smoking on more than one day in the past 30 days were classified as current smokers, and those who reported lifetime smoking, while not having smoked in the past 30 days, were classified as ever smokers; the others were classified as never smokers^15^.

Never-smokers were asked to respond to two validated questions to determine their susceptibility to smoking in the future: ‘Do you think you will smoke a cigarette in the next 12 months?’ and ‘If your best friend offers you a cigarette, may you test it?’. Participants were classified as susceptible if they did not respond ‘definitely no’ to both questions, while the others were classified as non-susceptible.

### Independent variables

Exposure to anti-tobacco messages was measured by the question ‘Have you noticed any information describing the dangers of smoking in the following channels and places within the past 30 days: a) traditional media (e.g. newspapers/magazines, television, films, broadcasting, notice boards), b) internet-based media (e.g. cell phone, computers, digital magazines), c) mobile digital billboards on buses or subways, d) billboards indoors (e.g. supermarkets or shop stores), and e) sport events, exhibitions, concerts, community assembly or community activities?’.

Exposure to pro-tobacco messages was also measured by the above-mentioned question, except the exposure message was the brand or tobacco advertisement, and one more question: ‘When you watch TV, video or movies, have you seen actors smoking?’.

Those who answered ‘no contact’ or ‘never seen’ scored 0, those who replied ‘sometimes seen’ scored 1, while those who answered ‘always seen’ scored 2. We calculated the total scores and classified students as ‘no exposure’ if the total score was 0, and others were classified as ‘exposure’. Adolescents were also categorized into the following groups: a) only exposure to anti-tobacco messages, b) only exposure to pro-tobacco messages, c) both, or d) neither. In order to investigate the effects of different exposure levels, we trisected the exposure level into ‘high level’, ‘medium level’ and ‘low level’, according to the total score.

### Covariates

Participants reported their gender, age, grade level, type of school, residence, boarding situation, grade point average (GPA), monthly pocket money, and school district. Parents smoking were classified into three categories: ‘none or do not know’, ‘one parent smokes’, and ‘both parents smoke’^16^. Friends’ smoking was classified as: ‘none’, ‘some of them’, and ‘the majority or all of them’. Depression was measured by the Chinese version of the Patient Health Questionnaire 2-item (PHQ-2-C), which assessed changes in their interest and mood over the past two weeks^17,18^. Participants with score ≥3 were classified as depressed^17^.

### Data analysis

To take the complex survey sample design into account, SPSS 22.0 software (IBM, Armonk, NY, USA) was used to perform statistical analyses. Sample characteristics and smoking-related characteristics were summarized using weighted percentages and confidence intervals. Percentages and chi-squared test were used to compare exposure to tobacco messages between different genders and types of school. Adjusted odds ratios (AORs) with 95% confidence intervals (CIs) of the associations between exposure to tobacco messages and current smoking as well as susceptibility to smoking were estimated by multivariate logistic regression model after controlling covariates (such as personal characteristics, depression, friends’ smoking and parents’ smoking) at baseline and stratifying by gender and type of school. A p-value <0.05 was considered statistically significant.

## RESULTS

### Descriptive statistics

As shown in Table 1, the overall sample was composed of students who attended junior (61.99%), senior (23.67%) or vocational (14.34%) high schools in Shanghai. The majority of the students were local residents (72.21%) and externs (86.45%). Over half had at least one parent who was a smoker (63.44%); less than 20% had friends who smoked (17.21%); and more than 10% were categorised as depressed (12.45%). For smoking status, 92.12% of the students were never smokers, 5.36% were ever smokers, and 2.52% were current smokers, while 7.67% of the students showed smoking susceptibility.

**Table 1:** Baseline characteristics and tobacco use among adolescents in Shanghai, China, 2017 (N=12278)

		*Weighted*		*Unweighted*
	
	*%*	*95% CI*	*Number*	*n (%)*
**Age**				
(mean years, 95% CI)	14.28	14.23–14.31	670050	14.62 (14.60–14.97)
**Type of school**				
Junior high school	61.99	61.13–62.85	415377	6462 (52.63)
Senior high school	23.67	22.88–24.48	158593	2475 (20.16)
Vocational high school	14.34	13.86–14.83	96080	3341 (27.21)
**District**				
Urban	33.36	33.13–33.60	223534	4042 (32.92)
Suburbs	66.64	66.40–66.87	446516	8236 (67.08)
**Gender**				
Male	51.60	50.69–52.52	345778	6419 (52.28)
Female	48.40	47.48–49.31	324272	5859 (47.72)
**Residence**				
Local	72.21	71.39–73.02	483864	8755 (71.31)
Non-local	27.79	26.98–28.61	186186	3523 (28.69)
**Boarding in school**				
Yes	13.55	13.00–14.12	90791	2302 (18.75)
No	86.45	85.88–87.00	579260	9976 (81.25)
**Monthly allowance**				
<200	61.23	60.35–62.11	173882	7011 (57.10)
200–600	25.95	25.17–26.75	85865	3401 (27.70)
>600 RMB	12.81	12.24–13.41	670050	1866 (15.20)
**GPA**				
Top 25%	32.96	32.11–33.83	220860	3924 (31.96)
Average	46.28	45.38–47.20	310129	5712 (46.52)
Bottom 25%	13.45	12.84–14.09	90136	1645 (13.40)
Not sure	7.30	6.85–7.78	48925	997 (8.12)
**Parents’ smoking**				
None	36.56	35.68–37.44	244962	4377 (35.65)
Some	59.25	58.35–60.14	396985	7364 (59.98)
Most or all	4.19	3.85–4.57	28103	537 (4.37)
**Friends’ smoking**				
None	82.79	82.13–83.43	554740	9693 (78.95)
Some	15.16	14.55–15.79	101550	2274 (18.52)
Most or all	2.05	1.83–2.31	13760	311 (2.53)
**Depression**				
No	87.55	86.94–88.13	586624	10688 (87.05)
Yes	12.45	11.87–13.06	83426	1590 (12.95)
**Smoking status**				
Never	92.12	91.64–92.57	617244	11091 (90.33)
Ever	5.36	4.98–5.76	35893	780 (6.35)
Current	2.52	2.28–2.80	16913	407 (3.31)
**Susceptibility to smoking**				
No	92.33	91.86–92.78	618651	11140 (90.73)
Yes	7.67	7.22–8.14	51399	1138 (9.27)

CI: confidence interval. GPA: grade point average. RMB: renminbi, official currency of the People’s Republic of China.

### Exposure to anti-tobacco and pro-tobacco messages

As presented in Table 2, the overall exposure rates of pro-tobacco and anti-tobacco messages were 89.9% and 91.5%, respectively. Urban school students were more prone to be exposed to high-level anti-tobacco messages than suburb school students (42.3% vs 36.7%; p<0.001), while female students were more susceptible than male students (39.9% vs 37.2%; p<0.001), and students in junior high schools were more prone compared with those in senior and vocational high schools (42.3% vs 33.3% and 31.0%, respectively; p<0.001). With regard to high-level protobacco messages, the results showed the opposite trend: suburb school students were more susceptible to be exposed than urban school students (37.5% vs 33.3%; p<0.001), male students were more prone than female students (36.5% vs 35.7%; p=0.007), and vocational school students were the most likely to be exposed, followed by senior and then junior high school students (46.1% vs 37.4% and 33.3%, respectively; p<0.001).

**Table 2:** Anti-smoking and pro-smoking information of adolescents in different districts, genders, and schools in Shanghai, China, 2017 (N=12278 )

	*District*				*Gender*			
	
	*Urban*	*Suburbs*	*χ^2^*	*p*	*Male*	*Female*	*χ^2^*	*p*
**Anti-exposure**								
No	8.6 (7.7–9.5)	8.5 (7.9–9.1)	0.01	0.919	10.8 (10.0–11.6)	6.1 (5.6–6.8)	84.03	<0.001
Yes	91.4 (90.5–92.3)	91.5 (90.9–92.1)			89.2 (88.4–90.0)	93.9 (93.2–94.4)		
**Pro-exposure**								
No	10.2 (9.3–11.2)	10.1 (9.5–10.8)	0.02	0.897	11.2 (10.4–12.0)	9.0 (8.3–9.8)	15.76	<0.001
Yes	89.8 (88.8–90.7)	89.9 (89.2–90.5)			88.8 (88–89.6)	91.0 (90.2–91.7)		
**Anti-exposure**								
Low	27.2 (25.8–28.6)	30.7 (29.7–31.7)	37.06	<0.001	32.4 (31.2–33.6)	26.4 (25.3–27.6)	53.42	<0.001
Medium	30.6 (29.1–32.0)	32.6 (31.6–33.7)			30.4 (29.2–31.5)	33.6 (32.4–34.9)		
High	42.3 (40.7–43.8)	36.7 (35.6–37.8)			37.2 (36.0–38.5)	39.9 (38.7–41.2)		
**Pro-exposure**								
Low	38.2 (36.7–39.8)	31.7 (30.6–32.7)	52.92	<0.001	32.6 (31.4–33.8)	35.2 (34.0–36.5)	10.68	0.007
Medium	28.5 (27.1–30.0)	30.8 (29.8–31.8)			30.9 (29.8–32.1)	29.1 (27.9–30.3)		
High	33.3 (31.8–34.8)	37.5 (36.5–38.6)			36.5 (35.3–37.7)	35.7 (34.4–36.9)		

	*High school*				
	
	*Junior*	*Senior*	*Vocational*	*χ2*	*p*
**Anti-exposure**					
No	7.3 (6.7–7.9)	10.0 (8.9–11.2)	11.6 (10.5–12.7)	24.64	<0.001
Yes	92.7 (92.1–93.3)	90.0 (88.8–91.1)	88.4 (87.3–89.5)		
**Pro-exposure**					
No	10.4 (9.7–11.2)	9.3 (8.2–10.4)	10.4 (9.4–11.4)	1.83	0.164
Yes	89.6 (88.8–90.3)	90.7 (89.6–91.8)	89.6 (88.6–90.6)		
**Anti-exposure**					
Low	27.4 (26.3–28.5)	32.0 (30.2–33.8)	34.7 (33.1–36.3)	35.16	<0.001
Medium	30.3 (29.2–31.4)	34.7 (32.9–36.6)	34.3 (32.8–35.9)		
High	42.3 (41.1–43.5)	33.3 (31.5–35.2)	31.0 (29.5–32.6)		
**Pro-exposure**					
Low	36.4 (35.3–37.6)	31.2 (29.4–33.0)	27.1 (25.6–28.6)	33.86	<0.001
Medium	30.2 (29.1–31.4)	31.4 (29.6–33.3)	26.8 (25.4–28.4)		
High	33.3 (32.2–34.5)	37.4 (35.5–39.3)	46.1 (44.4–47.8)		

	*Total*
**Anti-exposure**	
No	8.5 (8.0–9.0)
Yes	91.5 (91.0–92.0)
**Pro-exposure**	
No	10.1 (9.6-10.7)
Yes	89.9 (89.3-90.4)
**Anti-exposure**	
Low	29.5 (28.7–30.4)
Medium	31.9 (31.1–32.8)
High	38.5 (37.7–39.4)
**Pro-exposure**	
Low	33.9 (33.0–34.7)
Medium	30 (29.2–30.9)
High	36.1 (35.2–37)

### The impacts of exposure to anti-tobacco and pro-tobacco messages

Table 3 reveals the frequency-risk relationships between tobacco message exposure and current smoking status. Exposure to medium (AOR=1.58; 95% CI: 1.10–2.26) and high (AOR=1.98; 95% CI: 1.39–2.81) pro-tobacco messages was positively associated with prevalence of current smoking (p-trend<0.001) compared with low exposure, while a negative relationship was found between exposure to anti-tobacco messages and current smoking (AORmedium=0.60; 95% CI: 0.44–0.82; AORhigh=0.54; 95% CI: 0.39–0.76; p-trend<0.001). After stratifying by gender and type of school, we found this trend remained among male students and junior high school students by level of tobacco message exposure, regardless of type, as shown in Table 3.

**Table 3:** Relation of exposure level to current smoking in adolescents stratified by gender and type of school (AOR, 95% CI)* , Shanghai, China, 2017, (N=12278 )

	*Current smoking*	*Total*	*Gender*		*High school*		
	*% ( 95% CI)*		*Male*	*Female*	*Junior*	*Senior*	*Vocational*
**Anti-exposure**							
Low	4.3 (3.7–5)	ref=1	ref=1	ref=1	ref=1	ref=1	ref=1
Medium	2.1 (1.7–2.5)	0.60 (0.44–0.82)	0.62 (0.43–0.89)	0.61 (0.32–1.13)	0.45 (0.21–0.96)	0.65 (0.29–1.44)	0.81 (0.57–1.17)
High	1.5 (1.2–1.9)	0.54 (0.39–0.76)	0.54 (0.37–0.80)	0.54 (0.28–1.02)	0.41 (0.22–0.77)	1.07 (0.52–2.21)	0.47 (0.31–0.72)
p-trend		<0.001	0.002	0.068	0.003	0.882	0.001
**Pro-exposure**							
Low	1.5 (1.2–1.9)	ref=1	ref=1	ref=1	ref=1	ref=1	ref=1
Medium	2.7 (2.3–3.3)	1.58 (1.10–2.26)	1.92 (1.24–2.97)	0.96 (0.49–1.87)	2.71 (1.17–6.25)	1.51 (0.69–3.29)	1.08 (0.70–1.66)
High	3.3 (2.8–3.8)	1.98 (1.39–2.81)	2.48 (1.62–3.80)	1.17 (0.61–2.25)	2.96 (1.34–6.56)	1.96 (0.82–4.67)	1.44 (0.95–2.18)
p-trend		<0.001	<0.001	0.686	0.01	0.197	0.043
**Anti- by pro-exposure**							
Pro-exposure only	7.3 (5.4–9.7)	ref=1	ref=1	ref=1	ref=1	ref=1	ref=1
Anti-exposure only	0.9 (0.5–1.7)	0.48 (0.23–0.98)	0.41 (0.16–1.02)	0.52 (0.16–1.73)	0.17 (0.03–1.06)	N/A	1.69 (0.68–4.18)
Pro- and anti- exposure	2.2 (1.9–2.5)	0.64 (0.41–0.99)	0.78 (0.45–1.36)	0.40 (0.20–0.79)	0.44 (0.19–1.01)	0.69 (0.32–1.52)	0.99 (0.57–1.71)
No exposure	7.0 (5.1–9.5)	1.00 (0.52–1.92)	0.98 (0.43–2.20)	1.06 (0.33–3.41)	1.03 (0.26–4.05)	0.75 (0.22–2.57)	1.48 (0.69–3.18)

AOR: adjusted odds ratio, CI: confidence interval. *Multivariate logistic regression model adjusted for age, gender (female/male), type of school (junior/senior/vocational high school), district (urban/suburbs), boarding (local residents/externs), residence (local/non-local), GPA (top 25%/average/bottom 25%/not sure), pocket money (<200/200-600/>600 RMB), depression(yes/no), friends’ smoking (none/some/most or all) and parents’ smoking(none/some/most or all).

The same situation was true with regard to the susceptibility to smoking: among never smokers, those who reported moderate (AOR=0.50; 95% CI: 0.39–0.64) or high (AOR=0.37; 95% CI: 0.28–0.48) exposure to anti-tobacco messages were less likely to be susceptible to smoking, and those who reported moderate (AOR=1.74; 95% CI: 1.31–2.31) or high (AOR=2.63; 95% CI: 2.00–3.47) exposure to pro-tobacco messages had a higher susceptibility to smoking compared with those who were exposed at a low-level (Table 4). This association was observed in both genders and all school types. We found that the mentioned association was stronger in male than female students when exposed to moderate (AOR: 1.69 vs 1.53) and high (AOR: 2.45 vs 2.30) levels of pro-tobacco messages. Junior high school students were the most vulnerable students to pro-tobacco message exposure, while senior high school students were the most susceptible students to anti-tobacco message exposure.

**Table 4:** Exposure level and risk of susceptibility to smoking in adolescents stratified by gender and type of school among never smokers (AOR, 95% CI)*, Shanghai, China, 2017 (N=12278)

	*Susceptibility*	*Total*	*Gender*		*High school*		
	*% ( 95% CI)*		*Male*	*Female*	*Junior*	*Senior*	*Vocational*
**Anti-exposure**							
Low	2.3 (1.9–2.8)	ref=1	ref=1	ref=1	ref=1	ref=1	ref=1
Medium	4.1 (3.5–4.8)	0.50 (0.39–0.64)	0.54 (0.39–0.74)	0.48 (0.32–0.71)	0.53 (0.35–0.81)	0.38 (0.23–0.60)	0.69 (0.48–0.98)
High	5.4 (4.7–6.1)	0.37 (0.28–0.48)	0.42 (0.31–0.58)	0.34 (0.23–0.52)	0.42 (0.29–0.62)	0.27 (0.16–0.47)	0.45 (0.30–0.67)
p-trend		<0.001	<0.001	<0.001	<0.001	<0.001	<0.001
**Pro-exposure**							
Low	2.3 (1.9–2.8)	ref=1	ref=1	ref=1	ref=1	ref=1	ref=1
Medium	4.1 (3.5–4.8)	1.74 (1.31–2.31)	1.69 (1.18–2.42)	1.53 (0.98–2.39)	2.20 (1.40–3.47)	1.23 (0.74–2.04)	1.78 (1.12–2.82)
High	5.4 (4.7–6.1)	2.63 (2.00–3.47)	2.45 (1.74–3.46)	2.30 (1.51–3.52)	3.04 (1.93–4.77)	1.95 (1.17–3.22)	2.86 (1.88–4.34)
p-trend		<0.001	<0.001	<0.001	<0.001	0.017	<0.001
**Anti- by pro-exposure**							
Pro-exposure only	7.8 (5.7–10.6)	ref=1	ref=1	ref=1	ref=1	ref=1	ref=1
Anti-exposure only	1.1 (0.5–2.1)	0.21 (0.09–0.45)	0.28 (0.11–0.70)	0.10 (0.03–0.36)	0.24 (0.06–0.87)	0.18 (0.04–0.79)	0.32 (0.10–1.03)
Pro- and anti- exposure	3.9 (3.6–4.3)	0.65 (0.46–0.93)	0.67 (0.43–1.04)	0.62 (0.33–1.16)	0.88 (0.48–1.62)	0.46 (0.24–0.86)	0.79 (0.44–1.43)
No exposure	4.2 (2.6–6.5)	0.48 (0.26–0.90)	0.47 (0.22–1.01)	0.53 (0.18–1.60)	0.82 (0.30–2.25)	0.24 (0.07–0.88)	0.48 (0.20–1.20)

AOR: adjusted odds ratio, CI: confidence interval. * Multivariate logistic regression model adjusted for age, gender (female/male), type of school (junior/senior/vocational high school), district (urban/suburbs), boarding (local residents/externs), residence (local/non-local), GPA (top 25%/average/bottom 25%/not sure), pocket money (<200/200-600/>600 RMB), depression(yes/no), friends’ smoking (none/some/most or all) and parents’ smoking(none/some/most or all).

### Interaction between anti-tobacco messages and pro-tobacco messages

As shown in Table 3, among current smokers, adolescents exposed to only pro-tobacco messages represented the highest smoking rate (7.3%, reference group). Teenagers exposed to both pro-tobacco and anti-tobacco messages appeared to be 36% less likely to be current smokers compared with the reference group (AOR=0.64; 95% CI: 0.41–0.99, p<0.001). Teenagers exposed to anti-tobacco messages alone were more than half less likely to be current smokers (AOR=0.48; 95% CI: 0.23–0.98). The risks among those who were exposed to anti-tobacco messages significantly decreased among female students (AOR=0.40; 95% CI: 0.20–0.79) and junior high school students (AOR=0.44; 95% CI: 0.19–1.01) when simultaneously exposed to pro-tobacco messages.

Among never smokers, adolescents exposed to only pro-tobacco messages represented the highest rate of susceptibility to smoking (7.8%, reference group) (Table 4). Compared with the reference group, teenagers exposed to both pro-tobacco and antitobacco messages appeared to be 35% less susceptible to start smoking (AOR=0.65; 95% CI: 0.46–0.93), which was significantly observed in senior high school students (AOR=0.46; 95% CI: 0.24–0.86). Teenagers exposed to anti-tobacco messages only, were the least susceptible to start smoking (AOR=0.21; 95% CI: 0.09–0.45), regardless of gender or type of school.

## DISCUSSION

To date, a limited number of studies have concentrated on the relationship between smoking and tobacco-related message exposure in terms of both exposure level and its mutual influence. Our study made up for the deficiency of Chinese research in this field, revealing that the risk of current smoking and susceptibility to smoking in never smokers increased as the exposure level of pro-tobacco information increased, while that level decreased when exposed to further tobacco control messages. We also observed a 35% decrease in both risks among students receptive to both anti-tobacco and pro-tobacco messages compared with those who were only exposed to pro-tobacco messages.

The exposure of tobacco promotional advertisements to adolescents remains a serious public health problem over decades, despite the release of the World Health Organization Framework Convention on Tobacco Control (WHO FCTC) in 2003^11^. China ratified the FCTC in 2005; however, the implementation progressed slowly^19,20^. The present research showed that overall exposure among youth to pro-tobacco messages reached 89.9%, and more alarming among male, suburb school and vocational school students. These findings may be partly attributed to the smoking norms in China, in which smoking is overwhelmingly a male phenomenon^19^. Higher exposure in suburb school students may be attributed to differences in tobacco prevalence^21^ and environment due to weak implementation of smokefree policies in suburb areas^22^. Our finding targeting vocational school students could be explained by their special learning and living environment; they directly enter into the workforce after graduation and are more likely to encounter social smokers^23^. Fortunately, our study also revealed that more than 90% of adolescents reported having noticed anti-cigarette information during the last month, which was higher than the proportion reported in China (77.9%)^24^, Indonesia (71.69%)^25^ and the United States (57.9%)^13^. It is undeniable that China has put remarkable efforts on tobacco control in recent years^26^. Collectively, these findings suggest that the control of tobacco advertising and the dissemination of tobacco control information still need to be strengthened, especially among male, suburb school and vocational school students.

Tobacco industries take advantage of advertisements to arouse adolescents’ interest in tobacco use^27^, through shaping their perception of smoking norms, and perceived benefits and smoking risks^6^ as well. Previous studies demonstrated that exposure to point-of-sale displays was associated with 1.6 and 1.3 times higher odds of experimental smoking and susceptibility to smoking, respectively, among adolescents^9,28^. Our study has come to similar conclusions and revealed that the risks vary with exposure levels. This trend was also observed in a study conducted in Germany^29^. One possible explanation is that adolescents were more interested in tobacco products when exposed to more promotions that encourage them to try smoking, leading to susceptibility to subsequent smoking behaviours^30^.

Tobacco control messages have been proven to reduce youth smoking initiation, although there are a limited number of studies^31^. Tobacco control advertisements mainly use graphical images or individual stories to demonstrate the serious health effects of smoking. For example, the United States^12^ and Turkey^11^ both reported that exposure to antitobacco advertisements could strengthen youth nonsmoking intentions and perceptions of smoking harms (OR=1.25; 95% CI: 1.11–1.42), and decrease the probability of them becoming smokers (OR=0.74; 95% CI: 0.63–0.88)^12^. This correlation was also confirmed in the present study. Since the associated risk further decreased when adolescents had more exposure, we recommend greater tobacco-control message publicity under many circumstances, for more gains.

In addition, the interaction between tobacco control and promotional information was uncertain until recently^10,13,14^. There are currently two general opinions, one is that anti-tobacco messages would be unable to counteract the effects of tobacco advertising despite its independent protective effect^14^, and another is simultaneous exposure to an anti-tobacco message could reduce the probability of smoking initiation among adolescents already exposed to tobacco advertising^13^. Our findings support the latter opinion that tobacco control messages may influence the effectiveness of receptivity to cigarette advertising, with more than 30% reduction on the risk of both current smoking and susceptibility to smoking, suggesting an important window of opportunity to offset the impacts of tobacco promotion and advertising. Tobacco control messages make youth less receptive to cigarette advertising, and therefore reduce their smoking willingness and impulsive purchasing that may progress into more intensive and frequent smoking^32^.

In terms of adolescents’ current smoking, the association was more pronounced among male and junior high school students, regardless of the type of tobacco message exposure. Given that this population has a dominant smoking rate^33^, both increased anti-tobacco and reduced pro-tobacco messages could have notable benefits on male students. For junior high school students, their immature physiology and psychology may result in higher sensitivity to both tobacco promotion and tobacco control information. Among all populations of never smokers, the association between tobacco-related messages and susceptibility to smoking was more significant. As smoking initiation is one step to regular smoking^25^, the significance of preventing youth from smoking their first cigarette is particularly of great importance. Considering that both reduction of tobacco promotional advertising and the increase of tobacco control messages could never diminish smokers’ intention to smoke, future tobacco control efforts should mainly focus on the regulation of tobacco-related information.

In addition to traditional media, the internet-based platforms and social media have created new channels, in which tobacco industries can promote their products. The present online advertisements are targeting young people with no experience of smoking^7^. In order to reduce smoking rates and denormalise smoking among youth, online advertising and promotions need further comprehensive restrictions. Exploring effective methods and forms of tobacco control messages targeting adolescents has become significantly important. Future studies should focus on developing specific tobacco control programs for adolescents (i.e. considering released messages through new media channels, including the internet to gain more attention from adolescents).

### Limitations

This study has some limitations. Our cross-sectional research design precluded causal inference. Longitudinal studies are therefore required to examine causal relationships between tobacco-related message exposure and smoking behaviours. The respondents in our study were adolescents in urban areas of China; thus, our results may not reflect rural areas.

## CONCLUSIONS

Exposure to tobacco promotional and control messages was found to be common among adolescents, which was associated with their susceptibility to smoking and behaviors. Comprehensiveness and enforcement of bans on tobacco promotion and advertisement need to be implemented. Since tobacco control information can not only independently reduce the risk of smoking but also effectively offset the effects of pro-tobacco advertising, we recommend its wider application. Future studies should explore the effective forms and approaches related to tobacco control among adolescents.
